# Imaging-based chromatin and epigenetic age, ImAge, quantitates aging and rejuvenation

**DOI:** 10.21203/rs.3.rs-3479973/v1

**Published:** 2023-11-07

**Authors:** Martin Alvarez-Kuglen, Delany Rodriguez, Haodong Qin, Kenta Ninomiya, Lorenzo Fiengo, Chen Farhy, Wei-Mien Hsu, Aaron Havas, Gen-Sheng Feng, Amanda J. Roberts, Rozalyn M. Anderson, Manuel Serrano, Peter D. Adams, Tatyana O. Sharpee, Alexey V. Terskikh

**Affiliations:** 1 Sanford Burnham Prebys, La Jolla CA 92037, USA; 2 UCSD, Department of Physics, La Jolla, CA 92093, USA; 3 Salk Institute for Biological Studies, La Jolla, CA 92037, USA; 4 UCSD School of Medicine, 9500 Gilman Drive, La Jolla, CA 92093, USA.; 5 University of Wisconsin, Madison, WI 53705, USA; 6 The Scripps Research Institute, La Jolla, CA 92037, USA; 7 Institute for Research in Biomedicine (IRB Barcelona), Barcelona 08028, Spain; 8 Barcelona Institute of Science and Technology (BIST), Barcelona 08028, Spain; 9 Altos Labs, Cambridge Institute of Science, Granta Park CB21 6GP, UK

## Abstract

Biomarkers of biological age that predict the risk of disease and expected lifespan better than chronological age are key to efficient and cost-effective healthcare^1–3^. To advance a personalized approach to healthcare, such biomarkers must reliably and accurately capture individual biology, predict biological age, and provide scalable and cost-effective measurements. We developed a novel approach – image-based chromatin and epigenetic age (ImAge) that captures intrinsic progressions of biological age, which readily emerge as principal changes in the spatial organization of chromatin and epigenetic marks in single nuclei without regression on chronological age. ImAge captured the expected acceleration or deceleration of biological age in mice treated with chemotherapy or following a caloric restriction regimen, respectively. ImAge from chronologically identical mice inversely correlated with their locomotor activity (greater activity for younger ImAge), consistent with the widely accepted role of locomotion as an aging biomarker across species. Finally, we demonstrated that ImAge is reduced following transient expression of OSKM cassette in the liver and skeletal muscles and reveals heterogeneity of in vivo reprogramming. We propose that ImAge represents the first-in-class imaging-based biomarker of aging with single-cell resolution.

## INTRODUCTION.

With a steady increase in average lifespan and population aging^4,5^ measurement of the aging process is becoming increasingly important, underscoring the need for accelerated adoption of biomarkers of functional, also called biological, age^6^. Such biomarkers should quantitate disease and mortality risks better than chronological age, thus improving the efficacy and quality of geriatric care while reducing associated cost^7–10^. Biomarkers of aging include grip strength and gait^11^, frailty indices^12,13^, frailty-based clocks^14^, metrics of the immune system^15,16^, telomere length^17^, glycosylation readouts^18^, levels of cellular senescence^19^.

The fascinating discoveries of Hannum^20^ and Horvath^20,21^ have identified CpG sites in human blood and other tissues with consistent age-dependent changes in DNA methylation (DNAm), enabling the development of DNAm clocks. This discovery represents a major step in the development of aging biomarkers rooted in epigenetics. Since the original discovery, many other clocks have been established^22–26^, often utilizing functional biomarkers alongside DNAm readouts, yielding biological clocks such as PhenoAge^26^ and DunedinPACE^27^ tailored to specific cell types or pan tissues^28–31^. These biomarkers correlate with mortality^32,33^, may inform on the lifespan (GrimAge)^24^, and are sensitive to lifespan-altering interventions^34,35^. However, separating the biological components from the chronological components remains a chellenge^28,36^.

One common characteristic of all DNAm clocks is the use of linear regression^37^ on chronological age and, for biological clocks, surrogate functional biomarkers (e.g. plasma protein levels). It often takes large cohorts to build DNAm clocks; that is, to extract the most informative CpGs and their regression coefficients. To use these clocks, additional individual DNAm levels are sequenced and the clock CpGs are multiplied by the regression coefficients. Age acceleration/deceleration (biological age) is then calculated by comparing the position of the new sample with respect to the clock’s average (regression line). Note that such an approach de facto excludes the biological components of newly tested samples, which do not participate in building DNAm clocks. This is important because comparing the biological functions of an individual, which is highly context-dependent, with a “golden mean” could be less informative for a particular individual^38–40^.

Epigenetic alterations represent one of the primary characteristics of aging, as chromatin structure is intimately related to gene expression and regulation and, thus, cellular function^41^. Likewise, the loss of epigenetic information has been proposed to be a cause of mammalian aging^42–44^, and transient expression of Yamanaka’s OSKM factors^45^ is sufficient for shifting aged mice towards an apparently younger state in at least some organs an tissues^43,46^. Well-defined cell identities (defined by the global epigenetic state in a cell) ensure organismal homeostasis^47,48^. Although driven by master regulator transcription factors^49–51^, epigenetic changes may precede a global change of cell identity at the transcription or proteome level^52^; the new cell identity is associated with a new epigenetic state^53–55^. Unsurprisingly, the alteration of epigenetic states is generally associated with cellular functional and phenotypic changes^52–56^, including aging^44,57^. It is known that epigenetic changes during aging result in altered epigenome and chromatin accessibility, aberrant gene expression, reactivation of transposable elements, and genomic instability^58,59^. Which specific epigenetic marks best convey age-dependent alterations is unclear; however, several studies linked aging to the loss of heterochromatin and alterations in global and local levels of H3K9me3, H3K27me3, H4K20me3, and H3K4me3^58,60,61^. The pattern of active enhancers, marked with a combination of H3K27ac & H3K4me1^62^, is also age-dependent^63,64^.

Since these epigenetic modifications exist on the chromatin manifold, the information collectively encoded by their spatial arrangement will likely be relevant in determining the progress of epigenetic aging. Several years ago, we pioneered microscopic imaging of epigenetic landscapes rooted in the analysis of chromatin topography in single cells^65^. We employed immunolabeling with antibodies specific for histone modifications (e.g. acetylation and methylation marks) and automated microscopy to capture cell-specific patterns using image texture analysis, resulting in multiparametric signatures of cellular states^65^. Here, we took advantage of this technique to develop image-based chromatin and epigenetic age (ImAge), an approach to studying aging that is fundamentally different from other methods, such as DNAm clocks.

We discovered the emergence of age-associated trajectories as an intrinsic and principal property of the spatial chromatin evolution with time. We observed that ImAge correlates with chronological age in mouse peripheral blood mononuclear cells (PBMCs) and several solid organs without linear regression. Encouragingly, we discovered ImAge measurements were consistent with expected perturbations to biological age: observing that calorie restriction decreased ImAge and chemotherapy treatment increased ImAge. Critically, ImAge of skeletal muscles from chronologically identical mice inversely correlated with their locomotor activity, suggesting its utility as a biomarker that measures behavioral and functional differences. Finally, we demonstrated that ImAge is reduced following transient expression of OSKM cassette in the liver and skeletal muscle and reveals heterogeneity of in vivo reprogramming.

## RESULTS.

### Microscopic imaging of epigenetic landscapes.

**Fig. 1.**
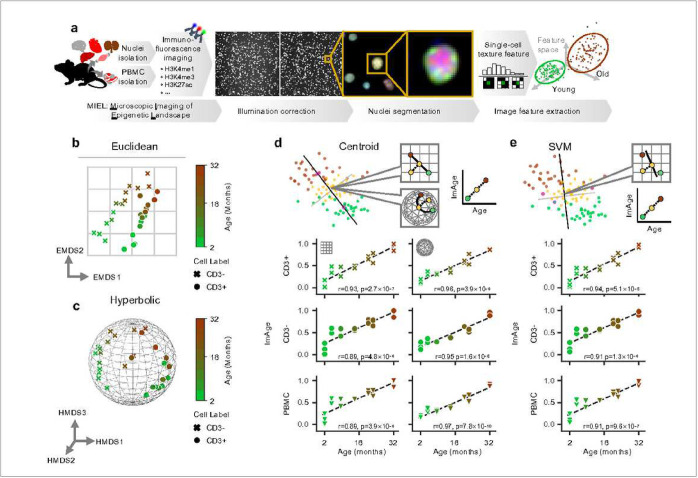
Emergence of chromatin trajectories of aging. **a**, Graphical representation of the MIEL workflow: Nuclear isolation, immunofluorescence imaging, image preprocessing, nuclear segmentation, texture feature extraction, and downstream analysis. **b-c**, ImAge calculations and regression analysis of CD3+ and CD3− subsets of PBMCs from C57BL/6NJ males aged from 1.7 to 32 months (1.7, 2.2, 5.3, 8.7, 15.1, 21, 22.3, 32.2) (n=2 per age group). **b**, 2-dimensional EMDS of texture features. **c**, 3-dimensional HMDS of texture features. **d-e**, Graphical representation of the method of ImAge axis construction above scatterplots of the resulting ImAge measurements versus age for the CD3+ subset (top), the CD3− subset (middle), and the whole population (PBMC) (bottom). **d**, ImAge using the geodesic connecting the centroids of the youngest and oldest groups in Euclidean or hyperbolic space. **e**, ImAge using Linear SVM fit to the youngest and oldest groups. PBMC: peripheral blood mononuclear cells. E/HMDS: Euclidean/hyperbolic multi-dimensional scaling, respectively.

We previously pioneered the technique of microscopic imaging of epigenetic landscapes, which captured patterns of nuclear immunostaining of epigenetic marks and derived multiparametric signatures in single cells^65^. Here, we applied this technique to investigate aging, with either freshly isolated primary cells or fixed isolated nuclei. Fig. 1a presents the schematics of microscopic imaging of epigenetic landscapes. In short, we used different combinations of histone post-translational modifications with well-recognized roles in aging (H3K9me3, H3K4me1, H3K4me3, H3K27me3, H3K27ac, H4K20me3)^58–63^ to label epigenetic marks (multiplexing subjected the availability of compatible primary antibodies). We used DAPI to label DNA/chromatin, which enables joint registration of all other epigenetic marks and provides chromatin pattern information. Acquired fluorescence images are processed using custom-built Python scripts, including nuclei segmentation with StarDist^66^, and feature extraction using threshold adjacency statistic (TAS)^67,68^ (Fig. 1a). It is important to note that, although the raw data are acquired from single cells (single nuclei), bootstrapped means (n=200 cells per bootstrap) were used to capture the properties of the single cell distribution while improving accuracy and equalizing the number of data points per sample. The size of the bootstraps was determined empirically by finding the lowest n while maximizing separation accuracy (**Extended Data Figs. 1 and 3**).

### The emergence of aging trajectories and construction of ImAge.

We assayed mouse PBMCs from C57BL/6NJ males aged from 1.7 to 32.2 months (1.7, 2.2, 5.3, 8.7, 15.1, 21, 22.3, 32.2 months). Cells were labeled with anti-H3K4me1, anti-CD3, and DAPI; image features were computed using H3K4me1, anti-CD3 and DAPI channels as described before^65^. We employed Euclidean multi-dimensional scaling (EMDS), to embed the data in a reduced 2-dimensional (2D) Euclidean space. EMDS revealed a clear separation of data points by cell type, CD3+ vs CD3−, mainly by the first dimension (EMDS1), and a clear age-related trajectory within each lineage, mainly by the second dimension (EMDS2) (Fig. 1b). The Kruskal’s stress1^69^ quantifies distortion of relative distances between data points when mapping from high (i.e., original TAS features 504 dimensions) to low (i.e., EMDS, 2 dimensions) dimensional space. A relatively small (0.08) EMDS Kruskal’s stress (Fig. 1b) confirmed strong preservation of relative distances in the data distribution. This strong preservation of distances implies the cell-type and age-associated trajectories observed in the EMDS are intrinsic principal characteristics of the epigenetic image texture features.

We sought out confirmation of the results observed above by using hyperbolic dimensionality reduction. Hyperbolic space has recently been shown to preserve properties of biological data distributions (i.e. relative distances between data points) in high-dimensional space better than Euclidean-based methods (e.g., EMDS)^70–73^. Thus, we sampled the CD3+/− single-cell data and embedded their relative distances into a 12-dimensional hyperbolic space with curvature (*κ* = 7.2) using hyperbolic multidimensional scaling (HMDS), optimized as previously described^70,74,75^. HMDS). Notably, the distances among samples when embedded in hyperbolic space exhibit significantly reduced distortions and uncertainties when compared to a Euclidean space with an equivalent number of parameters (dimensions): *R*^2^ = 0.99 for HMDS versus *R*^2^ = 0.67 for EMDS (**Extended Data Fig. 2c**). This observation substantiates the hypothesis that our data inherently adheres to a hyperbolic structure. We visualized this embedding in a three-dimensional (3D) hyperbolic space. Within this hyperbolic space, the shortest path connecting two data points is commonly referred to as a geodesic, which is curved instead of a straight line like in the Euclidean space (Fig. 1c). We observed a clear separation between CD3+ and CD3− cells, as well as the emergence of age-related trajectories for both CD3+ and CD3− subsets of the PBMCs (Fig. 1c). These findings confirmed the emergence of intrinsic aging trajectories within epigenetic image features of mouse CD3+/− subsets of PBMCs in two different geometries.

We aimed to find the simplest method to extract the observed aging trajectories. We reasoned the geodesic (the shortest path) connecting the centroid (average position) of the youngest and oldest provided the simplest solution. Measurement of epigenetic age can then be defined as the progression along this geodesic. We calculated the geodesic connecting the centroids of the youngest and the oldest mice in full dimensional space, which we will henceforth call the ImAge axis. We then calculated the progression along the ImAge axis via orthogonal projection, which we will henceforth call the ImAge (Fig. 1d, top graphics). Note that ImAge axes must be calculated separately for every experiment, as the relative distances of epigenetic image textures may vary between imaging conditions and tissues. Therefore, ImAge axes were constructed for CD3+ cells, CD3− cells, and PBMCS (all data) (Fig. 1d). In the Euclidean space, we observed a strong Pearson correlation of the centroid based ImAge with chronological age for CD3+ cells (r = 0.92, p = 2.7 × 10^−7^), CD3− cells (r = 0.88, p = 4.8 × 10^−6^), and total PBMCs (r = 0.89, p = 3.9 × 10^−6^) (Fig. 1d, left). In hyperbolic space, centroid-based ImAge provided notably stronger correlations with chronological age for all cell types: CD3+ cells (Pearson-R = 0.96, p = 3.9 × 10^−9^), CD3− cells (Pearson-R = 0.95, 1.6 × 10^−8^), and total PBMCs (Pearson-R = 0.97, 7.8 × 10^−10^) (Fig. 1d, right).

Critically, in both Euclidean and hyperbolic spaces, the distance between the youngest and the oldest mice along the ImAge axis was greater than the orthogonal distances to the axis (**Extended Data Fig. 2**). This indicated that changes along the ImAge axis are a principal source of variance in the dataset. Indeed, the variance along the ImAge axis accounts for a significant majority of the total variance: 81.5% for CD3+ and 79.1% for CD3−. Note that no regression on chronological age was necessary to reveal the aging trajectory: the trajectory is revealed as a principal feature of the dataset geometry, as seen in both Euclidean and hyperbolic embeddings (Fig. 1b and c). These properties indicated that the aging phenomenon is a principal source of chromatin and epigenetic texture variance in mouse PBMCs. For comparison, we analyzed a previously published mouse DNAm clock^76^ and found that the variance captured by the axis of linear regression is 0.15% (0.01% - 0.15%; 1^st^-99^th^ percentiles) of the total variance in the data (see Materials and Methods for details).

While the centroid-based method provided strong correlations with age, the centroid can be significantly affected by non-normal distributions in the reference groups (young and old) (Fig. 1d, top). This is not a desirable property given the heterogeneity of single-cell measurements, which may be composed of many cell types in various proportions, potentially leading to skewed or multimodal distributions. Therefore, to improve the robustness of the technique, we reasoned a modified approach that accounts for non-normal data distributions was in order. We utilized a linear support vector machine (SVM) to find the optimal geodesic regardless of the normality of the data distribution (Fig. 1e, top, see materials and methods). As before, all intermediate time points were projected onto the ImAge axis (Fig. 1d). The Pearson correlation of chronological age and ImAge using SVM was superior to that obtained from the original Euclidean centroid-based ImAge for all cell types: CD3+ cells (r = 0.94, p = 5.1 × 10^−6^), CD3− cells (r = 0.91, p = 1.3 × 10^−8^), and total PBMCs (r = 0.91, p = 9.6 × 10^−7^) (Fig. 1e and **Extended Data Fig. 1**). The unanimous improvement of accuracy in all cases conclusively supported SVM as a robust method for obtaining the optimal ImAge axis in Euclidean space.

Surprisingly, ImAge measurements from the SVM-based method were inferior to those obtained from the centroid-based hyperbolic ImAge. However, despite the higher accuracy, the computational cost of hyperbolic embedding limits the number of cells able to be embedded^70^. Therefore, we will use SVM to construct ImAge axes on all subsequent organs and tissues when operating in a Euclidean space, and when more precise measurements are required, we will use the original centroid-based method to construct the ImAge axis in hyperbolic space.

Taken together, these results suggest a spontaneous emergence of chromatin and epigenetic trajectories of mouse PBMC aging in both Euclidean and hyperbolic space. Critically, we described the ImAge axis connecting the youngest and oldest samples, which captures the principal (~80%) variance in the dataset.

### Comparative analysis of ImAge trajectories in solid organs.

Recent studies suggested that organs and tissues may age at a different pace in the same organism^77,78^. We investigated the distribution of ImAge readouts in 5 major organs, including brain, heart, kidney, liver, and skeletal muscles (quadriceps) in 3 cohorts of mice: young (2 months), middle age (15 months), and old (27 months). To directly compare ImAge trajectories in different organs and tissues, we developed a protocol to isolate nuclei from flash-frozen solid tissues (nuclei are PFA fixed immediately upon isolation to prevent any changes in chromatin state) and performed the imaging of chromatin and epigenetic landscape like that described for the freshly fixed cells (see Materials and Methods). We employed antibodies specific for H3K27me3, H3K27ac, H3K4me1 (+DAPI) to compute multiparametric image features as previously described^65^.

**Fig. 2:**
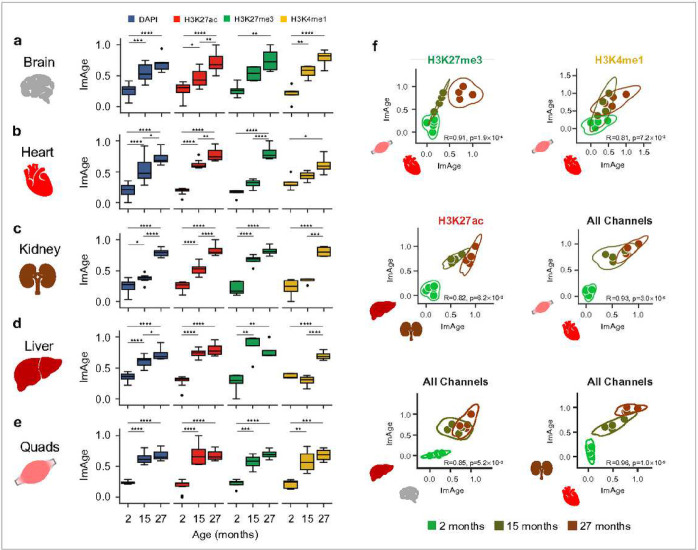
Age-related ImAge progression in multiple solid organs. **a-e** ImAge measurements and accuracy calculations on isolated nuclei from quadriceps (quads), liver, kidney, cardiac muscle (heart), and brain collected from three differentially aged cohorts of mice: 2 months (n=5) 15 months (n=4) and 27 months (n=4). Two plates were analyzed, both immunolabeled with H3K27ac+DAPI and then with either H3K27me3 or H3k4me1. Data for overlapping channels (DAPI & H3K27ac) were combined for computations. Boxplots minmax normalized 0–1 test set of bootstrapped data is shown. Differences of means were calculated via Tukey’s HSD. **f**, Consistent correlations were observed between skeletal muscle & heart (top row, bottom left), as well as brain & liver (bottom center), and heart & kidney (bottom right). Spearman’s R and p-values with Bonferroni correction for multiple comparisons. Significance values for all tests shown represent: * = p < 0.05, ** = p < 0.01, *** = p < 0.001, **** = p < 0.0001

A Euclidean ImAge axis was then constructed between the youngest (2 months) and oldest (27 months) samples for each organ separately. In all tissues and organs analyzed, we observed that ImAge was increased with chronological age (Fig. 2); however, the ImAge trajectories vary with epigenetic marks and organs tested (Fig. 2a–e). Separation accuracy between young and old samples was calculated per channel and for all channels combined (**Extended Data Fig. 3a-e**). Interestingly, the statistical significance of separation appeared to be mark-dependent. Pearson correlation of ImAge with chronological age yielded strong and significant (all p<0.05) correlations for brain (r = 0.84 ± 0.05), heart (r = 0.88 ± 0.03), kidney (r = 0.88 ± 0.06), liver (r = 0.81 ± 0.09), and skeletal muscles (r = 0.86 ± 0.04). Furthermore, among the 25 ImAge axes (4 epigenetic marks and all marks combined for 5 organs), 12 were most highly correlated with PC1, 10 for PC2 and 1 for PC3, with only 2 having no significant correlation with the first three PCs (heart-H3K27ac, kidney-all channels). Across the epigenetic marks (channels), taking the maximum Pearson correlation of ImAge and the first three PCs yielded strong correlations for skeletal muscle and kidney (r = 0.88 ± 0.08, 0.84 ± 0.10, respectively), moderate correlations for heart and brain (r = 0.88 ± 0.15, 0.84 ± 0.04, respectively) and a weak correlation for liver (r = 0.58 ± 0.15). All correlation values above are presented as mean ± standard deviation across the channels (**Supplementary Data Table 1**). These results extend our observation in blood to 5 solid organs and suggest a broad utility of ImAge to quantitate age-associated changes in chromatin and epigenetic organization. The predominant correlation with the first principal components indicates that organ aging is a principal source of chromatin and epigenetic texture variance measured by ImAge.

Next, we inquired about the relative pace of aging between the 5 different organs. Given the ubiquitous monotonic increase observed in all organs, we calculated the Spearman correlation between each pair of organs. We discovered a strong and robust correlation of ImAge in the heart and quads across several epigenetic marks and chronological age groups (Spearman-R: R=0.91, p=1.9 × 10^−4^, R=0.81, p=7.2 × 10^−3^ , R=0.93, p=3.0 × 10^−5^ for H3K27me3, H3K4me1, and all channels combined, respectively) (Fig. 2f). Other pairs correlated when all marks were combined (liver & kidney, heart & brain), but were not consistent across individual marks (Fig. 2f). There are likely additional significant and robust correlations to be further substantiated with a larger dataset and sample size (**Extended Data Fig. 4** and **Supplementary Data Table 2**).

Taken together, the strong and robust correlation observed between heart and skeletal muscle suggests we have observed organ-level synchronicity in the aging of two organs related by cell type (myocytes). The correlation observed between other pairs may indicate synchronicity that extends beyond cell type and will require further insights into the mechanisms.

### Age-related erosion of cellular epigenetic and information identity.

**Fig. 3.**
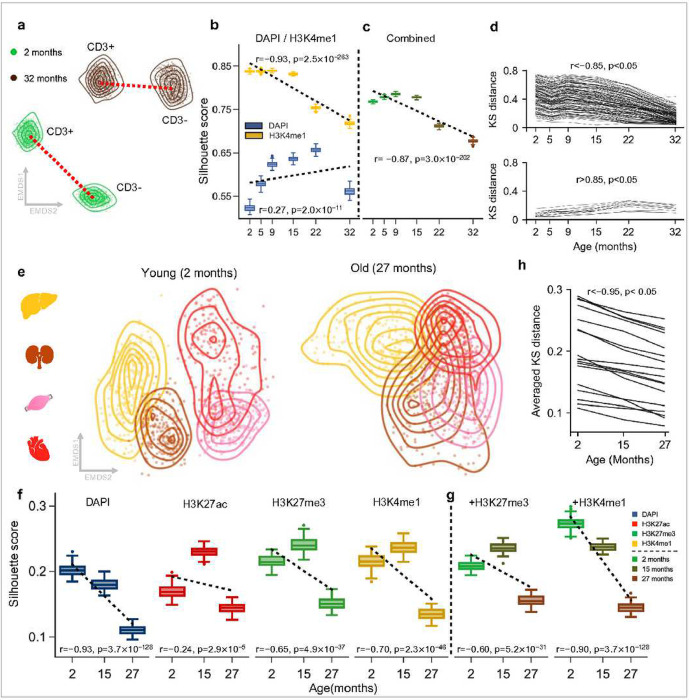
Age-related loss of cell type-specific chromatin and epigenetic information. **a**, A 2-dimensional EMDS of young (1.7 months) and old (32.2 months) CD3+ and CD3− subsets of PBMCs. b, c, Silhouette scores of CD3+ and CD3− subsets at indicated ages for individual marks (b) or their combination (c) using the information distance metric (based on mutual information and Shannon entropy, see Methods). **d**, the Kolmogorov-Smirnov (KS) distance analysis of CD3+ and CD3− subsets across indicated ages performed on significant features. **e**, A 2-dimensional EMDS of young (2 months) and old (27 months) liver, kidney, quads, and heart. **f**, **g**, Silhouette scores of 5 organs at indicated ages for individual marks (f) or their combination (g) using the information distance metric. **d**, the Kolmogorov-Smirnov (KS) distance analysis of 4 organs across indicated ages performed on significant features. Significant features were selected based on: 1) statistically significant (p < 0.05, Pearson |r|>0.85) KS distances between cell types and 2) a statistically significant (p < 0.05, Pearson |r|>0.95) correlation between KS distance and age.

From our lower-dimensional visualization of ImAge trajectories using MDS in both Euclidian and hyperbolic spaces, it visually appears that the separation between T cells (CD3+) and non-T cells (CD3−) is reduced with age (Fig. 1b and c). To quantify the age-related separation between CD3+ and CD3− we computed both information distances using two methods: the silhouette score (calculated across all features) and the Kolmogorov-Smirnov distance (KS distance) (see materials and methods for details). Briefly, the silhouette score measures the amount of overlap between multiple clusters of points, and the KS distance measures the difference between two distributions: a large silhouette score means less overlap and more separation between clusters, and a large KS distance means less overlap. The silhouette score revealed an age-related decrease in information distance between the CD3+ (T cell) and CD3− cells within PBMC samples (Fig. 3a–c), validating the visual assessment of MDS embeddings (Fig. 1b). The KS distance revealed a prevailing negative correlation between the separation of cell-type and age (127 out of 136 features), observed both for CD3+/CD3− cells (Pearson r < −0.85, p < 0.05) (Fig. 3d). These results indicate an age-related erosion of epigenetic identity in CD3+/− cell-types.

Intrigued by this discovery, we inquired if this decrease in information distance translated from cell types to organs. As with the blood data, we computed the silhouette score and KS distance between 5 organs: brain, liver, kidney, skeletal muscles, and heart. Remarkably, we observed a similar age-related decrease in information distance across the liver, kidney, heart, and quads (Fig. 3e), while the brain remained separated from the other organs (**Extended Data Fig. 5a**). Likewise, the KS distance measurements again revealed a prevailing negative correlation with age in liver, kidney, heart, and quads (Pearson r < −0.95, p < 0.05) (Fig. 3h) for the majority of significant features (17 out of 17 features). However, for the tissue samples, the number of significant features is comparatively fewer than in the blood (136 for blood vs 17 for tissue). This is likely due to the presence of only three distinct chronological age groups available for calculating the correlation between KS distance and age.

In sum, our analysis suggests an age-related erosion of cellular epigenetic identity and a loss of cell- and tissue type-specific information with the variable trajectories that depend on particular epigenetic marks and are organ and tissue-specific.

### ImAge tracks with the expected change in biological age.

**Fig. 4.**
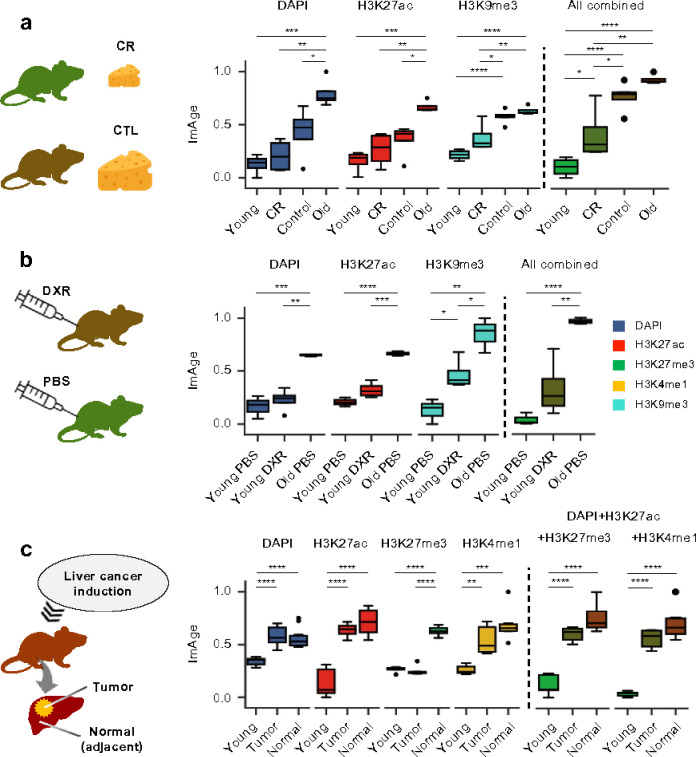
Diet, chemotherapy, and cancer affect ImAge. **a-c** ImAge calculations separated by epigenetic marks and several marks combined for indicated conditions for Caloric Restriction (CR), Doxorubicin (DXR), or induced hepatocarcinomas (tumor). **a**, young (3 mo., n=4) and old (24 mo., n=4) control mice were used to construct an ImAge axis upon which CR (7 mo., n=4) and control (7 mo., n=4) ImAge values were measured. **b**, young (1 mo., n=3) and old (27 mo., n=3) control mice treated with PBS were used to construct an ImAge axis upon which young DXR-treated mice (1 mo., n=4) ImAge values were measured. **c**, liver tissue from old mice (8 mo., n=3) with induced tumors was separated by the presence or absence of tumors. Normal old tissue along with young control mice (2 mo., n=3) were used to construct an ImAge axis upon which old tumor ImAge values were measured. In (c), two plates were analyzed, both immunolabeled with H3K27ac+DAPI and then with either H3K27me3 or H3k4me1. Data for overlapping channels (DAPI & H3K27ac) were combined for computations. Significance was calculated using Tukey’s HSD. All ages shown are in months. p-value cutoffs are as follows: *: 0.01 < p <= 0.05; **: 0.001 < p <= 0.01; ****: p <=.0001.

As a biomarker for aging, ImAge must not just correlate with chronological age, but distinguish perturbations to biological age. Therefore, we tested the capability of ImAge to measure several expected perturbations to biological age. Multiple studies suggest that calorie restriction (CR) slows aging in various species ^79–81^. We compared the effect of CR on ImAge of liver hepatocytes. C57BL/6J males were fed ad libitum (control) or 75% of ad libitum (25% CR diet) from 2 months of age. Nuclei were purified from frozen liver tissues as previously described ^82^, distributed in 384 well plates, fixed, and immunolabelled with anti-H3K9me3 and anti-H3K27ac (+DAPI). Images and multiparametric epigenetic image features were acquired as before and a Euclidean ImAge axis was constructed as described above. We observed that CR treatment shifts the ImAge readouts in liver hepatocytes towards that of a younger age (Fig. 4a). Statistical significance was driven by H3K9me3; with non-significant trends for H3K27ac and DAPI.

Previous work demonstrated the age-accelerating effect of widely used chemotherapeutic agents. We followed Demaria et al., 2017 protocol for doxorubicin treatment (10 mg/kg, i.p.; controls received PBS). Live hepatocytes were isolated 21 days post injection, to avoid acute stress response to DNA damage. Purified hepatocytes (2-step perfusion method) were plated in 384 well plates, fixed, immunolabelled with antiH3K9me3 and anti-H3K27ac (+DAPI), imaged, multiparametric image features were computed as before and a Euclidean ImAge axis was constructed as described above. We observed that doxorubicin treatment shifted the ImAge readouts from freshly isolated liver hepatocytes toward that of an older age (Fig. 4b). Statistical significance was again driven by H3K9me3; trend for H3K27ac and DAPI. Given that neither DAPI nor H3K27ac alone significantly separated either perturbation from controls, these data suggest the age-associated changes lie in differences in specific epigenetic modifications (H3K9me3) rather than in the overall chromatin structure.

Taken together these results suggest that ImAge, specifically, its H3K9me3-based component, tracks with the expected change of biological age in the liver cells following longevity interventions.

### ImAge of liver tumors and adjacent normal tissue.

We compared ImAge of liver tumors induced by diethylnitrosamine injection (postnatal day15) with normal tissues from the same livers in 8 months old C57BL/6NJ mice using immunolabeling for H3K27me3, H3K27ac, H3K4me1, and DAPI. No difference in ImAge was observed using DAPI or H3K27ac, whereas tumors appeared significantly younger with H3K27me3 (p <= 0.0001, Tukey’s HSD), and a trend was observed for H3K4me1(Fig. 4c and **Extended Data Fig. 6**). This suggests that the tumor state may appear as a return to a younger state for some specific epigenetic marks. However, these relationships do not dominate when all epigenetic marks are considered. In sum, these results that tumors may appear younger with respect to some epigenetic marks. The significance of this observation will need to be further elucidated.

### ImAge correlates with age-related behaviors in chronologically identical mice.

Next we investigated whether or not ImAge readouts map to functionally meaningful organismal readouts (which is to say, biological age) in chronologically identical samples. To this end, we conducted metabolic, cognitive, and motor behavioral tests in chronologically identical C57BL/6 males (25 months old, n=18) and acquired image features from the skeletal muscles. We used young (2 months old, n=5) and old (27 months old, n=5) mice to derive the ImAge axis of chronological young and old controls. We focused on skeletal muscles (quadriceps), which bear the functional load of motor behavior and mediate systemic metabolism^83^. Nuclei were isolated from flash-frozen tissues, immunolabeled with H3K27ac + H3K27me3 antibodies (+DAPI), imaged, and multiparametric epigenetic image features were acquired as previously described^65^.

**Fig. 5.**
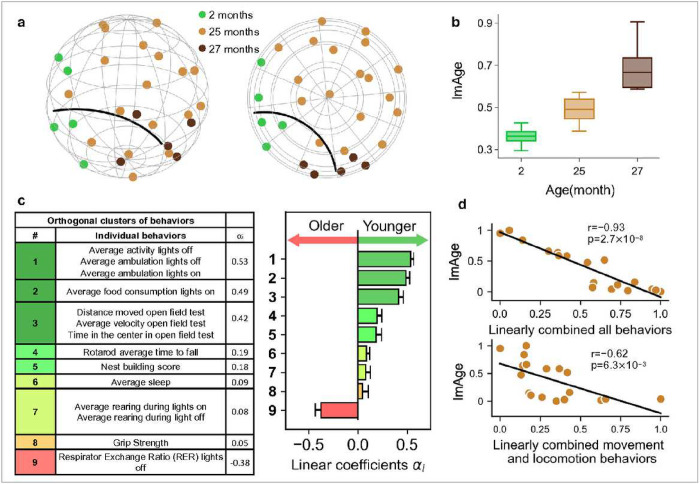
Locomotor activity is a salient correlate of ImAge in chronologically identical mice. **a**, A 3-dimensional representation of the 9-dimensional Hyperbolic embedding (HDMS) and its 2-dimensional projection (view from the top) of the young (2 months) and old (27 months) mouse quadriceps samples utilized as references to obtained centroids for the ImAge axis. Quadriceps samples from chronologically identical (25 months) mice from the behavioral cohort were co-embedded with reference mice to obtain (**b**) their ImAge distribution between the reference samples. **c**, the 9 orthogonal clusters of behavior with the coefficients for linear optimization correlating behavioral/functional readouts and ImAge. The direction of ImAge association with each cluster (older, younger) is proportional to the cluster’s α_i_. **d**, Correlations between ImAge and a linear combination of all behavioral readouts (top) and locomotor activities only, clusters 1, 3, 4, and 7 (bottom).

We embedded the data points, which are bootstrap means of 200 samples, into a significantly reduced 9-dimensional hyperbolic space using information distance as the metric. The performance in preservation of data structure using the HMDS (*R*^2^ = 0.87) evaluated with the shepherd diagram (**Extended Data Fig. 7a**) was significantly better than the EMDS (*R*^2^ = 0.35) using the same number of parameters (dimensions), supporting the notion that our data exhibits inherent hyperbolic structure. The curvature of this hyperbolic space was determined to be −8.4 (see materials and methods). The dimensionality and curvature were optimized as previously described^63,69^. We computed the geodesic between the centroids of young and old reference mice in the hyperbolic space and projected the coordinates of all mice onto this geodesic, resulting in a distribution of ImAge readouts. As with the other tissues, we observed a robust separation between the ImAge from young and old reference mice, and the ImAge readouts of experimental mice were distributed in between (Fig. 5a and b). The ImAge distribution on the aging geodesic effectively captures the substantial portion of data variance, accounting for 82% in the case of the chronologically identical mouse utilized for assessing behavioral performance (**Extended Data Fig. 7b**). This finding underscores the prominence of aging as the principal axis of variation within our dataset. Note that a 3-dimensional space in Fig. 5a is an imperfect visualization of a 9-dimensional hyperbolic space used for actual embedding. To extract relationships between ImAge readouts and behavioral readouts, we used linear regression. To ensure extracting the most stable relationships, we excluded non-significant, highly variable behavioral readouts (see materials and methods). To reduce collinearity in the regression analysis, we clustered correlated behaviors (**Extended data Fig. 7c** and **d**), thus deriving regression coefficients for 9 orthogonal clusters (Fig. 5c, see materials and method for details). The linear coefficient *α*_*i*_ is proportional to the contribution, positive or negative, of each cluster to ImAge. Because individual readouts are normalized and colinear within each cluster, we consider each of individual readouts within the cluster contribute to ImAge with the same coefficient *α*_*i*_. Such computations enabled us to identify key metabolic readouts and behaviors with positive or negative contributions to ImAge (Fig. 5c).

The linear combination of the 9 orthogonal clusters of metabolic and behavioral readouts provided excellent (Pearson r=−0.93 p=2.7 × 10^−8^) correlation with ImAge (Fig. 5d, top). This strong and highly significant correlation of ImAge with a relatively small number of whole organism metabolic and behavioral readouts underscores the potential utility of ImAge as a single biomarker of functional or biological age in skeletal muscles and, possibly, other tissues and organs. This linear combination of behavioral parameters can be conceptually framed as a weighted metric of behavioral performance. Notably, it reveals a substantial negative correlation with ImAge, signifying a discernible decline in behavioral performance with increased ImAge. Individually, clusters 2 and 3 significantly correlate with ImAge (**Extended data Fig. 7e**). Consistent with numerous previous studies, several forms of locomotor activity (cluster 1, 3, 4, 7) were negatively correlated with ImAge (greater activity for younger ImAge). Taken together these 4 major clusters provided strong (Pearson r=−0.62 p=6.3 × 10^−3^) correlation with ImAge (Fig. 5d, bottom). Respiratory Exchange Ratio (RER) during the dark phase (lights off) was positively correlated with ImAge (greater RER for older ImAge), in agreement with a recent cross-sectional study^84^.

We demonstrated the high efficiency of ImAge to capture the aging progression along the geodesic in the 9-dimensional hyperbolic space. We discovered that the variance along the geodesic (ImAge axis) accounts for 82% of the variance in this dataset. This is in line with the previously observed properties of hyperbolic space that appear to reduce the noise and better represent complex biological data^70,71,73^.

Taken together, these findings suggest that ImAge faithfully captures salient behavioral/functional readouts, such as locomotor activity, in chronologically identical animals, meaning that ImAge captures salient determinants of biological age.

### ImAge reveals heterogeneity of in vivo reprogramming with OSKM factors.

**Fig. 6.**
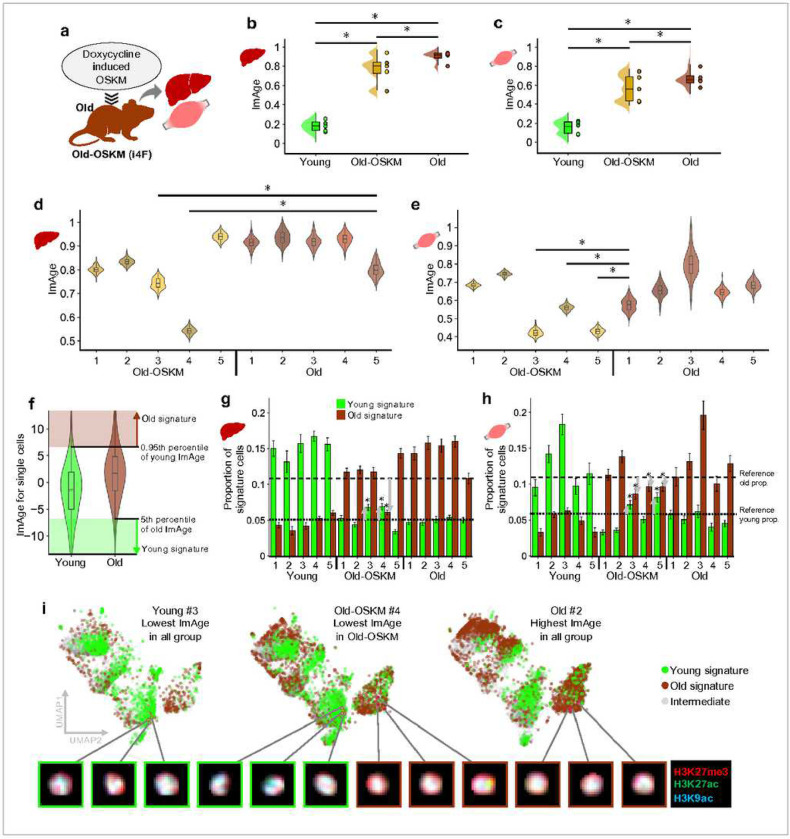
ImAge revealed heterogeneity of partial reprogramming in vivo. The chronological ages of young and old mice are 3.2 and 13.8 months, respectively. **a**, Evaluating the degree of reprogramming after doxycycline-induced OSKM factors (i4F mice, old-OSKM) in the liver and muscle. **b**, **c**, Distribution of ImAge (100 iterations of the test is shown) in the liver (b) and muscle (c). Dots on the right side of violin plots are the mean ImAge of individual mouse samples. **d**, **e**, Violin plots representing the distribution of the ImAge within individual samples in the liver (d) and muscle (e). Statistically significant differences were assessed between all old-OSKM samples and the old mice sample with the lowest ImAge (the “youngest” old mouse). **f**, Distribution of ImAge at a single-cell level. The mean accuracy of the young and old segregation was 0.620±0.001 (100 iterations). The young and old signatures are defined by the threshold of ImAge readout at 5th (and lower) and 95th (and higher) percentile values of old and young single cell ImAge readout, respectively. **g, h**, The proportion of young/old ImAge signatures in the liver (g) and muscle (h) for each animal. The gray arrows indicate an increase in young and a decrease in old signatures compared to the “youngest” old mouse defined in comparison of ImAge distributions (d and e). The dotted and dashed lines indicate the reference proportions of young and old signatures, respectively, in the “youngest” old mouse. **i**, Uniform manifold approximation and projection (UMAP) of single-cell texture features obtained from the liver samples. The green and brown data points represent the single cells with young and old ImAge signatures, respectively. The intermediate single cells are in grey. p<0.05 Mann-Whitney U-test.

We inquired whether ImAge reports the reversion of the aging process using OSKM-driven partial reprogramming in vivo^43,46^. We analyzed liver, heart, and skeletal muscle tissues from 13.8 month old i4F and littermate control mice treated for one week with a low dose of doxycycline (0.2 mg/ml)^46^. Nuclei were isolated from the frozen samples of young (3.2 months), old (13.8 months), and old treated with doxycycline to overexpress OSKM factors (old-OSKM) (Fig. 6a and **Extended Data Fig. 8a**, see ref. ^46^ for details of mouse samples and Materials and Methods for sample preparation). Nuclei were immunolabelled with H3K9ac, H3K27ac, H3K27me3, and DAPI, and confocal images were acquired with Opera Phenix (PerkinElmer). There were significant differences (Mann-Whitney U-test, p < 0.05) between the median ImAge of young and old samples in both tissues (Fig. 6b and c, and **Extended Data Fig. 8b** and **c**). We observed that the median ImAge in the old-OSKM group was significantly decreased compared to that in the old group, but was significantly higher than that of the young samples. These results suggest liver and muscle cells in old-OSKM mice are partially reprogrammed on average.

Mouse sample-wise heterogeneity in response to the reprogramming was observed in the comparison of age group-wise variance and sample-wise ImAge readout distributions. At age group comparison, the variance of ImAge in the old-OSKM group was significantly higher than that of young and old groups (Fig. 6b and c, and **Extended Data Fig. 8b** and **c**, Levene’s test, p < 0.05). In the liver sample, there were significant differences (Mann-Whitney U-test, p < 0.05) between the median ImAge of two (#3 and #4) among the five old-OSKM mice samples as compared to the mouse with the lowest median ImAge in the old group (old #5) (Fig. 6d). In the skeletal muscle, we observe significant changes of ImAge in three old-OSKM mice (#3, 4 and 5) (Fig. 6e). Almost no changes were detected in the heart consistent with the phenotypic observations^46^ (**Extended Data Fig. 8d**). These findings suggest that ImAge detects cellular rejuvenation and reports variable reprogramming efficiency, at least in the liver and muscle, between individual organisms.

We observed different degrees of partial reprogramming per epigenetic marks/channels. To evaluate the channel-wise difference, the ImAge axis was constructed in the same way as described above for each channel (H3K9ac, H3K27ac, H3K27me3, and DAPI). In the liver, strong partial reprogramming was observed using DAPI and H3K9ac (**Extended Data Fig. 9a, left**), where OSKM-old #1–4 showed a significant decrease in ImAge. In H3K27me3, OSKM-old mice #1, #3, and #4 were found to be significantly reprogrammed. H3K27ac showed the weakest impact; only one significantly reprogrammed mouse was found (OSKM-old #4). In the muscle, all channels showed almost equal strength of reprogramming with significant decreases of ImAge observed in two mice (OSKM-old #5 and #3/#4), except for H3K9ac, which showed a significant decrease in three mice (OSKM-old #3–5). DAPI and H3K27me3 had the same mice pair significantly reprogrammed (#3 and 5) (**Extended Data Fig. 9a, right**).

We leveraged single-cell-nature of imaging to get insights into the heterogeneity of reprogramming, which is of critical importance for rejuvenation^85^. We defined signature single-cells of the young and old groups and assessed the changes in the proportion of signatures in the old-OSKM group. We obtained ImAge readouts for single cells by measuring the location of single-cell features along with the ImAge axis constructed on binned data points from 200 cells (the ImAge axis used above). The signature cells for each age group (young or old) were defined by single cells, whose ImAge readout was hardly observed in the other group. For instance, young signature cells were cells with an ImAge readout lower than the 5^th^ percentile of old ImAge, meaning young cells can only be observed at a maximum of 5 percent in the old group (Fig. 6f). Conversely, the old signature cells were determined by ImAge readouts higher than the 95^th^ percentile of the young group (Fig. 6f, see Materials and Methods for details in determining the signature cells). We observed a significant increase in young signature cells and a decrease in old signature cells in the old-OSKM group on average (**Extended data Fig. 9b**) in the liver and a significant decrease of the old signatures in the muscle (**Extended data Fig. 9c**). We observed three different patterns of significant changes in the proportion of signature cells; an increase of young signature cells, a decrease of old signature cells and both of them. Particularly in the old-OSKM#4 in the liver, #3 and #5, which were three of the most reprogrammed mice, showed a significant increment of young signature and decrement of old signature (Fig. 6g and h). Because ImAge is rooted in the single-cell image analysis, cell nuclei with corresponding ImAge signatures can be identified (Fig. 6i and **Extended data Fig. 10**).

Taken together, these results suggest that ImAge is decreased in some of old-OSKM samples compared to old samples in liver and skeletal muscles, confirming the heterogeneity of in vivo reprogramming^46,86,87^. Further dissecting this phenomenon, ImAge uncovered reprogramming-associated changes of individual epigenetic marks and provided first insights into the dynamics of epigenetic-based cellular age in the reprogrammed samples.

## DISCUSSION.

Several major categories, including molecular, measuring specific molecules of omics, physiological, measuring physical performance and physical characteristics, and digital health, measuring wearable and non-wearable, biomarkers of aging have recently been proposed^6^. In addition, existing histologic, capturing tissue and organ morphology, biomarkers are less popular in aging research due to the measurement difficulties, tissue specificity, and scarcity of computational methods^6^. Here we describe novel class of biomarkers of aging, ImAge, based on imaging of chromatin and epigenetic patterns in single cells.

One of our most surprising findings is the discovery of intrinsic aging trajectories formed by multiparametric epigenetic image features that could be rendered and visualized using Euclidian and hyperbolic metrics. Remarkably, no regression was required for ImAge axis construction, yet all intermediate age groups were naturally arranged in a highly ordered manner. Serendipitously, the ImAge axis emerged as a principal source of information in the image data manifold. Given no prior expectation, this is a compelling property of ImAge, which indicates that we have extracted information proximal to an aspect of the aging phenomenon. We reason this aspect is related to the robust spatial organization of chromatin topography in individual nuclei in each organism and their evolution with time.

As an imaging-based approach, ImAge is complementary to previous aging biomarkers, such as DNAm clocks, which utilize a fundamentally different technique (sequencing). While ImAge is intimately linked with chromatin and epigenetic organization and is blind to sequence-level modifications, DNAm presents a sequence-level approach that is largely blind to the 3-dimensional organization of chromatin. Another notable difference between ImAge and DNAm clocks is the utilization of linear regression: DNAm clocks universally regress CpG methylation levels against chronological age (i.e. building DNAm clock) to construct robust population average useful for numerous applications^23,37^. DNAm clocks are based on a small subset of all CpGs in the genome (up to 1000 out of over 28M, or less than 0.01%). Although the actual variance of genome-wide DNAm is difficult to capture due to sample-wise variations in bisulfate sequencing, we estimated that rodent DNAm clocks extract from 0.15% (our estimate from publicly available mouse data) to 4.7%^88^ (rat DNAm clock) of the total variance in the DNAm dataset. In contrast, ImAge captures the data manifold’s major (~80%) characteristics, including in the case of chronologically identical mice (see below). We posit that the highly ordered nature of the image feature space results in our ability to perform direct and precise measurements of the relative distances between individual samples.

However, ImAge’s advantages do not come without limitation. DNAm clocks have become the gold standard for aging biomarkers, in part because they enable individual measurements to be easily compared against the population without reconstruction of entire model. A key part of the universality of these clocks is the stability of both DNAm itself and the biochemical technique to sequence CpGs. However, imaging is quite the opposite: image features are sensitive to imaging conditions (such as microscope setup and labeling procedures), leading to many opportunities for instability of feature values between experiments. Currently, this limitation demands that ImAge axes must be computed for each new sample combination. However, it is worth noting that the spatial organization of chromatin, the imaging substrate, although fundamentally variable at single cell level^89^ is likely not the issue. Rather the pixel values per channel and their relative intensities (from differences in antibodies, lasers intensities, detectors setup, etc). Future work should investigate the stability of the image-feature manifold across imaging conditions to separate biological variability at the single cell level from technical limitations. Fiduciary samples applied across multiple experiments and computational developments in deep learning provide the empirical path to addressing these questions.

Current single-cell DNAm analysis has been relying on statistical imputation due to sparsity of the methylation readouts for single cells^90^, limiting its practical applicability. For instance, exploration of intrinsic geometry using Euclidean and hyperbolic embedding could be challenging because it requires the same set of features to be compared for all single cells. While the image analysis presented here (mainly 20X magnification, 2-dimensional maximum projection, TAS features) struggles to fully resolve age-related chromatin changes at the single-cell level, we have only scratched the surface of the available imaging capabilities: high resolution confocal microscopy and advanced colocalization features present clear path towards extracting significantly more information from nuclear images and, potentially, highly structured single-cell resolution. Indeed, our accuracy measurements show near perfect separation accuracy at a single-cell resolution is not far away (**Extended data Figs. 1** and **3**). Thus, ImAge, being in its nescient stages of development, promises significant room for improvement on both imaging and computational fronts.

Imputing organ aging from plasma protein suggested different rates of organs and systems aging within the same organism (aging ageotypes)^78^ while the Tabula Muris Senis studies demonstrated a similar yet asynchronous inter-organ progression of aging^91^. Some degree of organ connectivity is plausible so that the decline of one organ can promote the dysfunction of other organs, accelerating organismal aging^92^. We observed an overall monotonic progression of ImAge along chronological age for all epigenetic marks and 5 organs tested. Together with our observations in PBMCs, it suggests that a monotonic correlation of ImAge and chronological age is universal. We observed a strong correlation of ImAge between the heart and skeletal muscles (quads) for all epigenetic marks (and DAPI) as well as the heart and kidney for all mark combinations. ImAge of the liver also correlated with the brain and kidney. Note that ImAge correlation between organs doesn’t mean that the pace of ImAge progression is the same in those organs. Incidentally, a metabolic imbalance of lipids accelerates inflammation and leads to lipotoxicity, mostly afflicting the kidney, heart, and skeletal muscle^93^, and lipotoxic insults precipitate aging^94^. Immobilization and atrazine induce oxidative stress in the liver, kidney, and brain, with curcumin and quercetin exhibiting a protective effect^95,96^.

Cellular epigenetic identity is integral to governing cell functionality and maintaining tissue homeostasis. We observed an age-related juxtaposition of cellular states and a diminished separation of cellular identity among diverse tissues or cells, suggesting an erosion of cellular chromatin and epigenetic identity. Although we documented this effect for CD3+ and CD3− cells in the blood and between 5 different organs, this is likely a general phenomenon. This could be explained by the age-related erosion of epigenetic and chromatin landscape^44^ which increases the noise in part through cell-to-cell variation in gene expression^97^, loss of lineage fidelity with age, and activation of lineage-inappropriate genes during aging^98–102^. Reactivation of human endogenous retroviruses during aging could also contribute to this process^103,104^. Our observations are consistent with the information theory of aging, which proposes that aging in eukaryotes is associated with loss of epigenetic information over time^105–107^ and that loss could be causative^44^.

DNA damage is known to induce cancer and accelerate aging ^108–110^ and chemotherapy treatments have damaging effects on the entire organism and accelerate the aging process ^111–114^ including increased frailty, chronic organ dysfunction, increase in cardiovascular diseases, cognitive impairment, and secondary cancers ^115–119^. We observed a shift of ImAge towards old age in mice treated with doxorubicin, resembling the observations in humans ^120^. The changes were driven by H3K9me3, suggesting possible changes in heterochromatin. If ImAge could faithfully report the effects of other chemotherapy drugs and in different organs, it will be useful to design interventions that increase the resilience to chemotherapy ^121^, and to reduce the aging effects of chemotherapy.

Because CR robustly increases maximum lifespan and delays biological aging in diverse scenarios ^79–81^, albeit the full picture could be more complex ^122,123^. successfully applied CR regimens help to understand the biology of aging ^124–126^ and could help recovery after chemotherapy in clinic ^121^. We have observed a shift in ImAge readouts in CR animals towards that of young animals consistent with the phenotypic observations. Again, the changes were driven by the H3K9me3 mark, which has been robustly associated with aging^88,127,128^ further substantiating the relevance of ImAge readouts for measuring biological age. It will be important to further refine these analyses, covering different CR regimens in mice and other species (e.g., monkeys), and to correlate the epigenetic changes with functional readouts at the organismal level as well as with various OMICs datasets (e.g., gene expression, chromatin accessibility, and enrichment for various epigenetic marks).

We observed younger ImAge in liver tumors, but only for H3K27me3 mark. Early studies suggested DNAm age acceleration in tumors compared to normal tissues^20,21,31^. However, recent studies matching tumor and normal tissue from the same organ/individual painted a more complex picture with deceleration of DNAm age in stomach adenocarcinoma^129^ and no change in triple-negative breast tumors^130^. Analysis of ~439 endometrial cancers suggested unchanged DNAm age in 90% of tumors while DNAm age deceleration was associated with advanced diseases and shorter patient survival^131^. Given that extended partial reprogramming in vivo results in tumors^132^, a younger chromatin and epigenetic age of tumors is rather intuitive.

We discovered that in chronologically identical mice, ImAge was inversely correlated with a weighted combination of behaviors. Indeed, a significant association between DNAm clock and physical function after controlling for age has been reported^88^. Here, the largest group of behaviors that inversely correlated with ImAge comprised various flavors of locomotor activity. Age-related decline in locomotor activity is a universal feature of living organisms well documented in Drosophila^133^ and insects in general^134^, rodents^135^, dogs^136^, primates^137^, and humans^138^ where it is associated with cognitive impairment and decline in brain dopamine activity^139^. Locomotion is a very reliable factor for exploratory behavior in mice^140,141^. The only correlate of older ImAge was the respiratory exchange ratio (RER), previously observed to be increased in old mice only in the dark phase^84^. Taken together, the nature of the correlated behaviors suggests that ImAge captures appropriate functional readouts to represent biological age. Further, ImAge captures 82% of the variance in this dataset comprising chronologically identical mice. Therefore, the principal aspect captured by ImAge represents the biological difference between animals, i.e., their biological age.

Finally, ImAge detected liver and muscle rejuvenation following one cycle of OSKM-mediated reprogramming in vivo. The advantage of analyzing a single cycle is that we detect the “proximal” effects of OSKM overexpression. With multiple cycles, the combined effect over months may include many secondary effects. Although the final outcome is more pronounced, the underlying mechanism is likely to be “buried” in many layers of effects over months. Curiously, different mice appeared to be mostly reprogrammed for liver and skeletal muscles, with DAPI and H3K9ac being the most informative to detect the change. Although we didn’t have H3K9me3 for this experiment the combination of DAPI and H3K9ac (complimentary to H3K9me3) suggests that we might be detecting heterochromatin changes. It will be informative to directly compare the performance of DNAm clocks and ImAge to quantify the apparent heterogeneity of in vivo reprogramming^142^. Given the robust correlation of ImAge with key physiological, behavioral, and metabolic metrics underlying functional decline with age, we posit that ImAge represents the first-in-class imaging-based biomarker of aging with single-cell resolution.

## MATERIALS AND METHODS.

### Mice

Experiments were conducted according to guidelines and protocols approved by the Institutional Animal Care and Use Committee (IACUC) of Sanford Burnham Prebys Medical Discovery Institute. Data presented within this manuscript were obtained using male mice. C57BL/6 mice, ranging in age from 2 to 27 months old, and were obtained from National Institute on Aging, Aged Rodent Colonies (RRID:SCR_007317).

### Mouse behavioral studies.

#### EchoMRI testing.

The EchoMRI 3-in-1 instrument (EchoMRI LLC, Houston, TX) is a quantitative nuclear magnetic resonance (qNMR) imaging system for whole body composition analysis of unaesthesized small animals ^143,144^, and qNMR body composition analysis with EchoMRI instrumentation has been proposed to be “gold standard” methodology for metabolic studies in the mouse ^145^. Following calibration, each mouse was put in a holder and placed into the EchoMRI chamber and lean mass, fat mass and water mass was calculated.

#### Optomotor test.

The optomotor allows for assessment of visual ability and consists of a stationary elevated platform surrounded by a drum with black and white striped walls. Each mouse is placed on the platform to habituate for 1 minute and then the drum rotates at 2rpm in one direction for 1 minute, is stopped for 30 sec, and then rotates in the other direction for 1 minute. The number of head tracks (15 degree movements at speed of drum) is recorded. Blind mice do not track the moving stripes.

#### Comprehensive Laboratory Animal Monitoring System (CLAMS).

Indirect calorimetry was performed in acclimated, singly-housed mice using a computer-controlled, open-circuit system (Oxymax System) that is part of an integrated Comprehensive Lab Animal Monitoring System (CLAMS; Columbus Instruments, Columbus, OH: ^146,147^). Testing occurred in clear respiratory chambers (20 × 10 × 12.5 cm) equipped with a sipper tube delivering water, food tray connected to a balance, and 16 photobeams situated in rows at 0.5 in intervals to detect motor activity along the *x*- and *z*-axes. Room air is passed through chambers at a flow rate of 0.5 L/min. Exhaust air from each chamber is sampled at 15-min intervals for 1 min. Sample air is sequentially passed through O_2_ and CO_2_ sensors (Columbus Instruments) for determination of O_2_ and CO_2_ content, from which measures of oxygen consumption (VO_2_) and carbon dioxide production (VCO_2_) are estimated. Outdoor air reference values are sampled after every 8 measurements. Gas sensors are calibrated prior to the onset of experiments with primary gas standards containing known concentrations of O_2_, CO_2_, and N_2_ (Airgas Puritan Medical, Ontario, CA). Respiratory exchange ratios (RER) were calculated as the ratio of carbon dioxide production (VCO_2_) to oxygen consumption (VO_2_). Energy expenditure measures (VO_2_, VCO_2_ and heat formation [(3.815 + 1.232*RER)*VO_2_ (in liters)]) were corrected for effective metabolic mass by using each mouse’s lean mass obtained from the EchoMRI test.

#### Open field test.

This test predicts how animals respond when introduced into a brightly illuminated open arena ^148^. It is a classical test of “emotionality” used to measure anxiety-like responses of rodents exposed to stressful environmental stimuli (brightly illuminated open spaces) as well as to capture spontaneous activity measures. The apparatus is a square white Plexiglas (50 × 50 cm) open field illuminated to 600 lux in the center. Each animal is placed in the center of the field and several behavioral parameters (distance traveled, velocity, center time, frequency in center) are recorded during a 5-minute observation period and analyzed using Noldus Ethovision XT software.

#### Novel object recognition test.

This test assays recognition memory while leaving the spatial location of the objects intact and is believed to involve the hippocampus, perirhinal cortex, and raphe nuclei ^149–151^. The basic principal is that animals explore novel environments and that with repeated exposure decreased exploration ensues (i.e., habituation; ^152^). A subsequent object substitution results in dishabituation of the previously habituated exploratory behavior (^152–154^) and is expressed as a preferential exploration of the novel object relative to familiar features in the environment. Mice were individually habituated to a 51cm × 51cm × 39cm open field for 5 min. They were then be tested with two identical objects placed in the field for 5 min. After two such trials (each separated by 1 minute in a holding cage), the mouse was tested in the object novelty recognition test in which a novel object replaced one of the familiar objects. Behavior was video recorded and then scored for object contact time. The first time the mice were tested the objects used were clear plastic rectangular boxes filled with blue marbles and green plastic drink bottles filled with water and for the second test the objects were amber glass bottles and glass flasks filled with beige marbles. All objects were too tall for the mice to climb up on and too heavy for the mice to move.

#### Footprint Pattern Test.

Basic gait measures can be assessed using simple footprint pattern analysis ^155,156^. Nontoxic paint was applied to each mouse’s paws (a different color was used for front and back paws). The mouse was then placed at one end of a runway covered in paper and allowed to ambulate until their paws no longer left marks. Measurements were forelimb and hindlimb stride lengths (left and right) and front and back leg stride widths. Three full strides were averaged for each mouse’s values. Data were excluded from mice that did not make 3 measurable strides (i.e. they circled or stopped).

#### Barnes maze test.

This is a spatial memory test ^157–159^ sensitive to impaired hippocampal function ^160^. Mice learn to find an escape chamber (19 × 8 × 7 cm) below one of twenty holes (5 cm diameter, 5 cm from perimeter) below an elevated brightly lit and noisy platform (75 cm diameter, elevated 58 cm above floor) using cues placed around the room. Spatial learning and memory were assessed across 4 trials (maximum time is 3 min) and then directly analyzed on the final (5^th^) probe trial in which the tunnel was removed and the time spent in each quadrant was determined, and the percent time spent in the target quadrant (the one originally containing the escape box) was compared with the average percent time in the other three quadrants. This is a direct test of spatial memory as there is no potential for local cues to be used in the mouse’s behavioral decision.

#### Grip strength test.

Grip strength was measured with a mouse Grip Strength Meter (Columbus Instruments) according to the manufacturer’s instructions (User Manual 0167–007). All-limb measurements were performed with the angled grid attachment, pulling the mouse towards the meter by the tail after engagement of all limbs. Four consecutive measurements per mouse were taken and the highest three values were averaged, and data were expressed as newtons of peak force.

#### Hanging wire test.

The hanging wire test allows for the assessment of grip strength and motor coordination ^161,162^. Mice were held so that only their forelimbs contact an elevated metal bar (2 mm diameter, 45 cm long, 37 cm above the floor) held parallel to the table by a large ring stand and let go to hang. Each mouse was given three trials separated by 30 seconds. Each trial was scored as follows and the average for each mouse was calculated: 0 — fell off, 1 — hung onto the wire by two forepaws, 2 — hung onto the wire by two forepaws, but also attempted to climb onto the wire, 3 — hung onto the wire by two forepaws plus one or both hindpaws around the wire, 4 — hung onto the wire by all four paws plus tail wrapped, 5 — escaped (crawled to the ring stand and righted itself or climbed down the stand to the table). Latency to falling off was also measured up to a maximum of 30 s.

#### Rotarod test.

Rotarod balancing requires a variety of proprioceptive, vestibular, and fine-tuned motor abilities as well as motor learning capabilities ^156^. An Accurotar rotarod apparatus (Omnitech Electronics, Inc., Columbus, OH) was used in these studies. A protocol was used whereby the rod starts in a stationary state and then begins to rotate with a constant acceleration of 10 rpm. When the mice were incapable of staying on the moving rod, they fell 38cm into a sanichip bedding filled chamber, breaking a photobeam in the process. The time of fall (translated to the speed at fall) was recorded by computer. The mice were tested in four sets of 3 trials, alternating directions between sets which were 30 min apart.

#### Treadmill test.

The treadmill exhaustion test evaluates exercise capacity and endurance ^163^. Mice are motivated to run to exhaustion in order to escape a shock at the base of the treadmill. Mice were trained to run in three daily 5 min sessions in which stopping would result in the mice touching the back of the apparatus and experiencing a mild shock (200 msec pulses of electric current with pulse repetition rate of 3 times per second (3 Hz) and an intensity of 1 mA). The treadmill speed for training was 10 m/min (0.373 mph). For the exhaustion test, the speed was initially set at 10 m/min for 5 min, and was increased 2 m/min every 2 min up to a maximum speed of 46 m/min (1.7 mph). The mice were run until they were exhausted or the maximal speed was achieved (which would mean a maximum run time of 41 min). Exhaustion was defined as the inability of the animal to run on the treadmill for 10 sec despite receiving shocks, a maximum of 30 mild shocks. To prevent injury, the mice were monitored carefully and continually during each session, and immediately upon meeting the criterion for exhaustion the shock grid was turned off and the mouse was removed from the treadmill.

### Isolating Nuclei from Frozen Tissues

Flash-frozen in liquid nitrogen (stored at −80°C) is a common practice to preserve tissue and organs that are not to be processed immediately ^164–166^. Organs and tissues were collected from freshly dissected mice, snap frozen using liquid Nitrogen, and stored at − 80°C. Organs and tissues were then transferred to a pre-chilled mortar and laid on top of dry ice; liquid Nitrogen was poured over the frozen tissue and a pestle was used to grind and pulverized the sample until a uniformly fine powder was obtained. Pulverized sample was the aliquoted and returned to − 80°C. To extract nuclei from frozen aliquots, 500 ul homogenization buffer (Nuclear Isolation Buffer 1 (NIM1) consisting of 250 mM Sucrose, 25 mM KCL, 10 mM Tris-buffer pH 8.0, 5 mM MgCl_2_, 1 mM DTT, and 10% Triton X-100) were added to powdered tissue and transferred to the mixture a glass Dounce homogenizer and dounced ~ 10 times (avoiding bubbles) on ice. Add homogenization buffer up to 1 mL and filter homogenization solution through a 40 mm cell strainer. Centrifuge filtered solution at 600×g (acceleration 4, deacceleration 4) for 4 min at 4° C. Aspirate supernatant and resuspend in 200 mL of PBS. Nuclei were then count on CellDrop FL (DeNovix) using 1:1 Acridine Orange /Propidium Iodide and homogenization solution. Samples were diluted in PBS to 1 million/mL to seed each well (~30,000 cells in 30 mL/well) of 384 well plate (Perking Elmer PhenoPlate 384-well black, clear flat bottom Cat No. 6057300) pre-coated (1 mL/25 cm^2^) with poly-L-Lysine (50 mL/mg). Centrifuge plate at 4000×g for 15 min at 4° C and immediately added 60 mL of 4% PFA to each well and incubate for 15 min at 4° C. Followed by one wash of PBS then proceed with microscopic imaging of epigenetic landscapes.

### Isolating Peripheral Blood Mononuclear Cells (PBMC’s)

200 mL of blood was collected retroorbital for each mouse and immediately mixed with an equal volume of 50 mM EDTA (to prevent coagulation). Blood mixture was further diluted with 200 mL of PBS and carefully layering on top of 750 mL of Ficoll-Paque Plus (Millipore Sigma Cat. No. GE17-1440-02); followed by density gradient centrifugation at 700×g for 30 minutes at room temperature. The PBMC rich layer (cloudy phase) was carefully collected avoiding mixing the above and below layers and transferred to a new tube containing 10 mL of PBS. The PBMC mixture was centrifuged at 700×g for 20 minutes at room temperature and then removed supernatant and resuspend pellet in 1 mL of PBS. Isolated PBMC’s were counted manually using a hemocytometer (Hausser Scientific Cat. No. 3120). Samples were diluted in PBS to 1 million/mL to seed each well (~30,000 cells in 30 mL/well) of 384 well plate (Perking Elmer PhenoPlate 384-well black, clear flat bottom Cat No. 6057300) pre-coated (1 mL/25 cm^2^) with poly-L-Lysine (50 mL/mg). Centrifuge plate at 4000×g for 15 min at 4° C and immediately added 60 mL of 4% PFA to each well and incubate for 15 min at 4° C followed by PBS wash.

### Doxorubicin treatment

Two months old C57BL/6 mice were intraperitoneal injected with doxorubicin (Santa Cruz Biotechnology, Cat. No. 25316-40-9) 10 mg/Kg. Doxorubicin was diluted in 150mM NaCl solution, and control mice were injected only with the vehicle solution (150mM NaCl). Fourteen days after treatment, mice were sacrificed and liver collected and immediately snap frozen in liquid nitrogen.

### Caloric restriction

Animal studies were conducted in accordance with approved protocols submitted through the respective Institutional Animal Care and Use Committees (IACUCs) at the University of Wisconsin Madison. Caloric Restriction mice: male C57BL/6N mice were individually housed under pathogen free conditions. Mice were randomized into control or restricted groups at 2 months of age and fed the AIN-93M semi-purified diet (Bio-Serv) either a Control diet (95% ad libitum) or Restricted diet (25% less than control). The mice were sacrificed at 7 months of age and tissues were harvested, flash frozen and stored at −80°C.

### Liver cancers

Animal studies were conducted in accordance with approved protocols submitted through the respective Institutional Animal Care and Use Committees (IACUCs) at the University of California San Diego. C57BL/6(?) male mice were intraperitoneally injected with 25 mg/kg diethylnitrosamine (DEN; N0258, Sigma-Aldrich) at postnatal day 15. At 8 months of age the mice were then sacrificed and dissected tumor and non-tumor parts of the liver and samples were immediately fresh frozen using dry ice/2-methylbutane bath and then stored at − 80 °C. All tissue collection occurred during a 4-hour time frame (Zeitgeiber time 7–11; corresponding to 1pm-5pm) to minimize circadian effects on metabolism, proliferation, etc.

### Microscopic Imaging of Epigenetic Landscapes

Wells were blocked with 2% BSA in PBS 0.5% Triton X-100 for 1 hour at room temperature, and then incubated with primary antibody overnight at 4C and then washed with PBS 3× (5 minutes each at room temperature). Next, wells were incubated with secondary antibody overnight at 4C and then washed with PBS 3× (5 minutes each at room temperature). Antibodies were used at the following concentrations: Anti H3K27ac 1:1000 (Active Motif Cat No. 39685), and /or Anti H3K27me3 1:1000 (Active Motif Cat No. 39155), and/or Anti H3K4me1 1:1000 (Active Motif Cat No. 61633). Antibodies were detected using the following secondary antibodies for their appropriate hots: Goat anti-Rabbit IgG (H+L) Alexa Fluor^™^ 488 (Thermo Fisher Scientific Cat. No. A11034), and Donkey anti-Mouse IgG (H+L) Alexa Fluor^™^ 488 (Thermo Fisher Scientific Cat. No. A31570). Wells were counterstained with DAPI (Thermo Fisher Scientific Cat. No. D1306) during the secondary antibody staining and plates were sealed with adhesive foil (VWR Cat. No. 60941–124). Cells/nuclei were imaged on either an Opera Phenix high-content screening system (PerkinElmer) or an IC200-KIC (Vala Sciences) using a 20x objective. At least five fields/well and a total of 9 z-stacks at a 1mm z-step for Opera Phenix and nine fields/well and a total of 10 z-stacks at a 1mm z-step for IC200 were acquired and five wells per mouse sample were imaged. Unless stated otherwise, at least three wells and a minimum of 300 cells for each condition were used for analysis.

### Image Feature Extraction

Image features were extracted for each single cell (nucleus) within a segmentation mask annotating cell nuclei on the images. The radial falloff ^167,168^ artifact of the images was corrected using BaSiC Illumination correction on all channels as a preprocessing step^167^. The segmentation was performed using StarDist^[Citation error]^ applied to DAPI images. We defined cell nuclei as the segmentation masks after the removal of small objects with an area corresponding to a radius less than 3–7*μm*, assuming spherical shape, dependent on tissue and microscope used. The cell nuclei masks were applied to all channels to isolate raw images of each nucleus in each channel. Texture features (252 2-dimensional TAS features; 756 3D TAS features per epigenetic mark/channel) were calculated from each nucleus for each channel as previously described^67,68^. In this study, 28 binarized images per epigenetic mark/channel were obtained by applying band-pass thresholding with the following intervals: $\left(v_{m},\infty \right),\;\left(v_{m}-v_{m}p,\infty \right),\;\left(v_{m}+v_{m}p,\infty \right),\;\left(v_{m}-v_{m}p,v_{m}+v_{m}p\right)$, where $v_{m}$ indicates mean pixel/voxel values within a cell nuclei mask, p indicates a factor to determine the width of the band, ranging from 0.1 to 0.9 with the step of 0.1. One static interval and three width-variable intervals with nine factors derive 28 binarized images.

### ImAge Axis Construction

The ImAge axis was obtained using z-scored features of young and old groups based on their centroids or linear SVM models. The centroid-based axis was then defined by the vector from the centroid of the young group to the centroid of the old group. The SVM-based axis was defined by the vector orthogonal to the hyperplane optimized to separate young and old groups; the precise location of this axis can be defined as the intersection of the orthonormal vector to the hyperplane and the centroid of all data points. The calculation of centroids and optimization of SVM models were performed using all features (without feature selection) over all data points resampled using bootstrap (see the following section for details of bootstrapping). It is important to note that the ImAge axis does not deform the feature space in any way; it is a simple linear transposition and projection.

The robustness of the ImAge axis was validated in 100 iterations of tests, where 75% and 25% of data were randomly sampled without replacement for training (axis construction) and testing (projection onto the axis), respectively. This training step only involves the young and old groups. Importantly, the other age or condition groups (middle age, perturbations) were not included in the training.

At each iteration of training/testing, we resampled single cell data with bootstraps of 200 cells to obtain the average data point representative to each mouse sample with uncertainty of the average. The bootstrap was performed 1000 times for each mouse sample to equalize the number of data points per mouse. In our analysis, the utilization of bootstrap sampling is essential to examine the aging related changes in the cell composition. Throughout the aging process, the cell identity remains unchanged, while the composition of the cell distribution undergoes variations. For instance, individual T cells from a young and an older mouse may appear identical, but when sampling a bootstrap of 200 cells from blood samples, the composition of cells, or the ratio between different cell types, differs between young and old mice. Clustering the single cells do not reveal the difference between young and old samples but simply put the cells of the sample type together.

The ImAge orthogonal distance was defined by the distance to the ImAge axis. Thus, the orthogonal component can be considered the positive scalar value representing the distance of a datapoint to the ImAge axis. The orthogonal distance $d_{o}$ for a data point can be given by $d_{o}=\sqrt{x^{2}-p^{2}}$, where x is the relative coordinate of the datapoint with the origin of the ImAge axis, and p is the coordinate of the same data point projected on the ImAge axis (i.e., $\sqrt{p^{2}}$ is the ImAge readout).

### Spearman correlation between multiple tissues

To select the most robust correlations, we removed 1 age group iteratively and corrected for multiple comparisons. Spearman correlations were calculated via pairwise comparisons between all combinations of either 2 or 3 age groups per organ pair per channel. P-values were post-hoc corrected for multiple comparisons using the Bonferroni method. An alpha of 0.05 was applied to the p-values and significant correlations per organ pair per channel were counted. Organ pairs which were correlated in both all age groups and 2 age groups with the 3^rd^ removed were considered robust and significant.

### Information distance metric

We have used a distance metric based on mutual information. Considering two random variables x and y from corresponding distribution X and Y that are normalized between 0 and 1 using min-max normalization, the mutual information I(X,Y) measures how much uncertainty of one variable is reduced by knowing the other variable. In other words, I(X,Y) measures the information shared by both variables. The variation of information D(X,Y) ^169^, or information distance metric we call here, is a measure of information that is not shared by both variables. Like the correlation and correlation distance, mutual information and information distance measures the dependence and independence between two random variables. However, unlike the correlation and correlation distance that measures the linear relationship between two variables, mutual information and information distance can capture the nonlinear relationship between two variables. The information distance can be written in terms of marginal entropies: H(X), H(Y), and joint entropy H(X,Y)

(1) $$D(X,Y)=\frac{H(X,Y)-I(X,Y)}{H(X,Y)}=\frac{2H(X,Y)-H(X)-H(Y)}{H(X,Y)}$$

In practice, we can consider two samples as x and y, with corresponding feature values $x_{i}$ and $y_{i}$ where $1\;\leq \;i\leq N_{\mathrm{feature}}$. and $N_{\mathrm{feature}}$. is the number of features. The probability density X and Y, can be approximated by the histogram p(z) of the feature values for each sample where p(z) is the number density of feature values $x_{i}$ or $y_{i}$ that sit inside the bin centered at z with bin size h=0.1. Entropy can be calculated using the Shannon entropy $H(z)=-\sum_{z\in Z}\;p(z)\;log(p(z))$ for $z=z_{0},z_{1}$.

### Embedding the data in the hyperbolic space

Hyperbolic geometry provides continuous approximation to tree-like hierarchical system, resulting in the distinguishing property of exponential expansion of states compared to quadratic expansion of states in Euclidean space ^170^. The hyperbolic geometry can be visualized using Poincare half space model and Poincare disk ^170^. Hyperbolic space is better suited compared to Euclidean space for representing complex multipara metrical biological datasets. This is because hyperbolic space enables using fewer dimensions, inferring the hidden nodes based on the activity of observed “leaf” nodes, and reading out the “centrality” of each node ^171–173^. Using the information distance metric, the nodes in the middle are features that sample take different values and the nodes on the boundary are samples. In another words, the sample is a representation of a serious feature value selection starting from middle to boundary.

Considering the aging process is due to a sequence of biological changes, and each change is chosen from a variety of choices, hence the number of possible states increase exponentially. We employed the Hyperbolic Multidimensional Scaling (HMDS) ^72^, which is a version of MDS in hyperbolic space. Euclidean MDS minimizes the difference between the distances calculated in the original Euclidean data space and the distances calculated in the high dimensional reduced space. Once we specify the dimension of the reduced space, the quality of embedding could be assessed using Shepherd diagram, which compares the distance in the original space and the distance in the dimensionality reduced hyperbolic space.

In order to quantify the performance of the dimension reduction using MDS, we can plot the Shepherd diagram where we compare the distances between samples in the original high dimensional space and the distances between samples in the lower dimensional embedded space. In general, the better the MDS performs, the closer the distances between samples embedded in the lower dimension correlate linearly with the distances between samples in the original space. The linear relationship ensures that the distances between samples are not distorted when we embed the samples from original high dimensional space to lower dimensional space. The correlation, the tightness of linear fitting, ensures the accuracy and robustness of the embedding.

### Finding linear coefficients for behaviors

We took advantage of the logarithmic and exponential representations, which map the points from the hyperbolic space to the tangent space centered at a reference point and vice versa ^174^. The tangent space is Euclidean, and the curved geodesic in the hyperbolic space can be approximated by a straight line in the tangent space which is easier to compute.

In our analysis, we consider n samples with distinct biological ages represented as the column vector $\vec{t}$ of dimension n. Similarly, we have N behavioral features, each denoted as ${\vec{f}}_{l}$, where i ranges from 1 to N, also column vectors of dimension n. Instead of assessing the correlation between ImAge $\left(\vec{t}\right)$ and individual readouts $\left({\vec{f}}_{l}\right)$, we explore the collective impact of these readouts on ImAge. We calculate the correlation R between ImAge $\left(\vec{t}\right)$ and the optimal linear combination of features, given by $\sum_{i}\alpha_{i}{\vec{f}}_{l}$, where $\left\{\alpha_{i}\right\}$ represents coefficients that maximize R, minimize p-values, and satisfy $\sum_{i}\alpha_{i}^{2}=1$ . Non-significant and highly variable features have been systematically filtered, with a specific focus on those whose p-value, in terms of correlation with the ImAge variable, exceeds the threshold of 0.3. These features have been removed from the analysis, ensuring a more rigorous and refined dataset for subsequent investigations.

To ensure the uniqueness of the linear coefficients $\left\{\alpha_{i}\right\}$, we compute a correlation matrix among all normalized significant feature vectors, grouping correlated feature vectors into orthogonal clusters. We identify 9 major orthogonal clusters of behavioral readouts, selecting a representative readout ${\vec{f}}_{l}$, from each cluster for coefficient computation. To estimate feature variance and ensure consistency across multiple measurements, we applied four trials of jackknife resampling to each ${\vec{f}}_{l}$, which encompasses several measurements over several days. Additionally, we conducted 1000 trials of reshuffling and recombination, such as selecting the first measurement from ${\vec{f}}_{l}$ and the second measurement from ${\vec{f}}_{J}$. The linear coefficients $\left\{\alpha_{i}\right\}$ are computed as averages over these trials for each orthogonal cluster ${\vec{f}}_{l}$.

### Separation between different cell types

To assess changes in epigenetic variation across diverse cell types during aging, we utilize two key metrics: the Silhouette score and the Kolmogorov–Smirnov distance (KS distance). The Silhouette score evaluates clustering performance, adaptable to both original and dimensional reduced data. In this study, we calculate Silhouette scores directly on raw data to avoid the bias introduced in the various dimension reduction processes. We utilize both the information distance and Euclidean distance metrics to illustrate changes in separation among different cell types from both an informational and a value perspective. Also, we employ 100 bootstraps to estimate the silhouette score within each age group, which allows us to assess the statistical stability and robustness of the silhouette score across various age categories.

To further confirm our observation of reduced separation between cell types during aging, we need a metric quantifying differences across cell types in individual features. The KS distance measures the distance between one-dimensional distributions characterizing distinct cell types. We sample 5000 cells per feature to capture distributions of specific cell types at given ages. Evaluating differences across $N_{\mathrm{tissue}}$ tissue types follows a systematic approach: pairwise KS distances are computed between each tissue pair, with subsequent averaging. The resulting average KS distance is given by $\sum_{i=1,j>i}^{N_{\mathrm{tissue}}}\;KS(i,j)/C\left(N_{\mathrm{tissue}},2\right)$ where i,  j denote cell types and the KS(i,j) is their KS distance and $C\left(N_{\mathrm{tissue}},2\right)$ is number of combinations choosing 2 pairwise tissues from total $N_{\mathrm{tissue}}$ tissues

### OSKM mice analyses and identification of young and old cell signatures

To compare each mouse at the individual level, the SVM model was trained using averaged data points obtained by bootstrapping 200 single-cell data points (see Methods for details of bootstrap sampling). The accuracy of the separation of young and old samples was 1.000 and 0.997 in testing for the liver and muscle samples, respectively, in 100 iterations of tests using random data splits (see Methods for details of training/testing scheme). We obtained ImAge based on the distance from the hyperplane of a linear SVM using 3D TAS features extracted from confocal images.

The signature cells for each age group (young or old) were identified as single cells where the ImAge readout was hardly observed in the other group and can be given by following indicator functions for young signature $I_{\mathrm{young}}$ and old signature $I_{\mathrm{old}}$;

(2) $$I_{\mathrm{young}}(a)=\left\{\begin{array}{l} 1,\;\mathrm{if}\;a<q_{\mathrm{old},5}\\ 0,\;\mathrm{otherwise} \end{array}\right.$$

and

(3) $$I_{\mathrm{old}}(a)=\left\{\begin{array}{ll} 1,\;\mathrm{if} & a>q_{\mathrm{young},95}\\ 0, & \mathrm{otherwise} \end{array}.\right.$$

Here, a indicates the ImAge readout for a single cell (nucleus), $q_{old,5}$ indicates the 5^th^ percentile value of single-cell ImAge readout in old samples, and $q_{\mathrm{young}\mathrm{,95}}$ indicates the 95th percentile value of single-cell ImAge readout in young samples. The proportion of signature cells and intermediate population can be obtained by calculating the proportion of them across all single-cell data obtained from each mouse sample. The choice of percentile values can adjust the purity of the definition of signature cells. For instance, using 0^th^ percentile (minimum value) of old in eq.(2) to define the young signature will ensure the selection of single cells only appear in the young group. In this study, we could not find clear boundary between young and old groups because of the heterogeneity of single cells; the single-cell ImAge readouts for young and old groups were largely overlapped (Fig. 6f). Hence, We determined the young signatures by selecting the 5th percentile, which accounted for only 5 percent of young signature cells in the old group, considering these 5 percent as statistical noise. Similarly, we adopted the opposite approach to determine the old signature. This approach ensured that we had a sufficient number of signature cells without compromising the quality of the data by including too much statistical noise.

### DNA methylation clock analysis

To compare ImAge to a comparable mouse DNA methylation clock, we selected the whole lifespan mouse multi-tissue (WLMT)^76^. First, reduced representation bisulfate sequence (RRBS) data collected from C57BL/6 whole blood samples of various ages were downloaded from GEO (accession number GSE80672). Only control mice were obtained for the purposes of comparison. Data preprocessing was performed as previously described with the following changes: newer versions of Trim Galore! (v0.6.1)^175^ and Bismark (v0.24.2)^176^ were used for adapter trimming and sequence alignment. Due to the sparsity in RRBS data versus our TAS features, CpGs with less than 99% coverage across all samples were eliminated for PCA for a fairer comparison between techniques. Any missing WLMT clock CpG sites after filtering were replaced. Total variance was calculated from the densified data, which was subsequently used to calculate the proportion of variance explained by the WLMT clock. To further equalize the comparison, DNAm data was randomly sampled without replacement (n=16, the same number of samples in our blood PBMC dataset) for 100 iterations and PCA and variance explained were computed for each iteration. Here we report the 1^st^-99^th^ percentile of these 100 iterations, and confirmed the total variance explained without subsampling is within these bounds.
